# Comparative analysis of knowledge representation and reasoning requirements across a range of life sciences textbooks

**DOI:** 10.1186/2041-1480-5-51

**Published:** 2014-12-18

**Authors:** Vinay K Chaudhri, Daniel Elenius, Andrew Goldenkranz, Allison Gong, Maryann E Martone, William Webb, Neil Yorke-Smith

**Affiliations:** SRI International, Menlo Park, CA 94025 USA; Monta Vista High School, Cupertino, CA USA; Cabrillo College, Aptos, CA USA; University of California, San Diego, CA USA; Foothill Community College, Los Altos Hills, CA USA; American University of Beirut, Beirut, Lebanon; University of Cambridge, Cambridge, UK

**Keywords:** Ontology, Textbook knowledge, Knowledge representation, Reasoning, Question answering, Semantic infrastructure

## Abstract

**Background:**

Using knowledge representation for biomedical projects is now commonplace. In previous work, we represented the knowledge found in a college-level biology textbook in a fashion useful for answering questions. We showed that embedding the knowledge representation and question-answering abilities in an electronic textbook helped to engage student interest and improve learning. A natural question that arises from this success, and this paper’s primary focus, is whether a similar approach is applicable across a range of life science textbooks. To answer that question, we considered four different textbooks, ranging from a below-introductory college biology text to an advanced, graduate-level neuroscience textbook. For these textbooks, we investigated the following questions: (1) To what extent is knowledge shared between the different textbooks? (2) To what extent can the same upper ontology be used to represent the knowledge found in different textbooks? (3) To what extent can the questions of interest for a range of textbooks be answered by using the same reasoning mechanisms?

**Results:**

Our existing modeling and reasoning methods apply especially well both to a textbook that is comparable in level to the text studied in our previous work (i.e., an introductory-level text) and to a textbook at a lower level, suggesting potential for a high degree of portability. Even for the overlapping knowledge found across the textbooks, the level of detail covered in each textbook was different, which requires that the representations must be customized for each textbook. We also found that for advanced textbooks, representing models and scientific reasoning processes was particularly important.

**Conclusions:**

With some additional work, our representation methodology would be applicable to a range of textbooks. The requirements for knowledge representation are common across textbooks, suggesting that a shared semantic infrastructure for the life sciences is feasible. Because our representation overlaps heavily with those already being used for biomedical ontologies, this work suggests a natural pathway to include such representations as part of the life sciences curriculum at different grade levels.

## Background

Using knowledge representation is now commonplace across a range of biomedical projects [1–3]. This usage is evidenced by the success of the National Center of Biomedical Ontologies, which, as of 2014, publishes and disseminates more than 350 ontologies [4]. Despite this widespread application of knowledge representation in biomedical projects, further significant value could be reaped: *The Journal of Nucleic Acids Research* catalogues thousands of databases that could substantially benefit if they were accompanied by an explicit ontology [5]. We anticipate that knowledge representation will play a crucial role in future biomedical research, especially for exploiting, leveraging, and understanding big data.

During an artificial intelligence (AI) project called Project Halo, we developed an intelligent textbook technology that leverages an explicit ontology and a question-answering system, and that helps students learn better [6]. Obvious overlaps exist between the technologies used in our project and the methods that are commonplace for biomedical ontologies [7, 8]. This convergence presents an unprecedented pathway for synergy between work on ontologies and life sciences education. If textbook knowledge could be represented and encoded in an educational context, as we propose here, then it could eventually be more widely incorporated into biomedical projects, thus complementing the existing knowledge resources.

Our work on the intelligent textbook [6] focused on an introductory college-level biology textbook called *Campbell Biology* [9]. We encoded substantial portions of *Campbell* and then used this knowledge representation as a basis for an intelligent textbook called *Inquire*, which enables students to explore topics across multiple levels of organization and to pose their own questions, which are then answered by machine reasoning. The intelligent textbook is a powerful learning tool that both gives students information such as definitions and descriptions of terms, and enables them to explore structure, function, and concepts across different levels of biological organization.

The current paper’s focus is on investigating the question: To what extent can a generic methodology for capturing textbook knowledge be developed that is applicable across a range of life sciences textbooks? We have broken this high-level question into three sub-questions: (1) To what extent is knowledge shared between the different textbooks? (2) To what extent can the same upper ontology be used to represent the knowledge found in different textbooks? (3) To what extent can the questions of interest for a range of textbooks be answered by using the same reasoning mechanisms? A desired outcome is quantifying the extent to which the already developed methods apply to different textbooks and quantifying any differences or novel requirements across textbooks. These questions are important, because if we could apply the same methodology to textbooks at both lower and higher grade levels, then this generalizability would enable making semantics integral to science textbooks. The answers to these questions will also be informative to others as they seek insights both into generic techniques for ontology design and into the requirements that differ across domains.

For the remainder of this section, we give an overview of our project, review the prior work on knowledge representation, describe the ontology, and provide the rationale for the textbooks that were selected for comparison. We follow that by a description of our methods and results.

### Context of Project Halo

Project Halo was an AI project funded by Vulcan, Inc., with the goal of creating a system called *Digital Aristotle* that could answer questions on a wide variety of science topics. SRI International participated in this project from 2003–2013 [6, 10, 11]. During this period, we advanced the state of the art in knowledge base (KB) systems by enabling domain experts with little background in knowledge representation to author knowledge that could be used for answering questions. This work’s results are embodied in a knowledge-authoring system called *AURA* [11]. To demonstrate the scalability of the approach, we used AURA to encode substantial fractions of *Campbell Biology* [9], which resulted in the knowledge base *KB Bio 101* [12]. A team of biologists trained in AURA but having no background in knowledge representation performed the encoding work. We designed a knowledge-factory process that the biologists used to systematically convert the textbook content into KB Bio 101 [12]. Although accurately assessing the total effort invested in the encoding is difficult, we estimate that the effort was at least twelve person years. KB Bio 101 represents a substantial fraction of *Campbell Biology* and contains more than 100,000 axioms [13].

We incorporated KB Bio 101 into an electronic textbook application called *Inquire*, which helps students with reading and homework problem solving [6]. An evaluation of *Inquire* with students showed the practical utility of incorporating a KB into an electronic textbook, as the *Inquire* students exhibited higher scores than did the control group and received no grades D or F, while these lower grades were seen in the control group. A video based on *Inquire* won the best video award at the annual conference of the Association for Advancement of Artificial Intelligence (AAAI) in 2012^a^.

### Knowledge representation in AURA

The AURA knowledge-authoring system uses *Knowledge Machine* (KM) as its knowledge representation and reasoning engine [14]. KM supports standard representational features such as classes; individuals; class-subclass hierarchy; disjointness; slots; slot hierarchy; necessary and sufficient properties; and deductive rules. The representation in KM can be formally understood as first-order logic with equality. Uniquely, KM’s representation supports graph-structured class descriptions. We illustrate KM in the following example.

Suppose we wish to represent the statement: “*Every cell is an entity that has a ribosome and a chromosome as its parts*”. We can express this statement in first-order logic as follows. (We implicitly assume that the statements hold over all times).

Axiom A1:


Next, suppose we wish to represent: “*Every eukaryotic cell has as parts a ribosome*, *a nucleus*, *and a eukaryotic chromosome such that the chromosome is inside the nucleus*^*b*^”. We can capture this statement in first-order logic as follows:

Axiom A2:


In the class definition of a *Eukaryotic*-*Cell*, specifying the **is**-**inside** relationship between the *Chromosome* and the *Nucleus* violates the tree model property [15]. In models satisfying tree model property, each node has (at most) a unique direct predecessor, and in general, it is a good indicator of decidability. To see how the valid models for A2 violate the tree model property, we create a directed graph as follows: each variable in the axiom is represented by a node, and a directed edge exists between the nodes representing a variable *x* and a variable *y* if they both participate in the same predicate such that *x* appears in the first position and *y* appears in the second position. (Because DLs are limited to binary predicates, we limit our discussion to only binary predicates.) For a graph for axiom A2, the node y_3_ has two incoming edges from *x* and y_2_, and thus, violates the tree model property. DL systems achieve decidable reasoning by limiting the representation to only allow tree models, and this limitation is well known [16]. Active research is in progress to address this limitation [17–20].

Next, suppose we wish to explicitly state the inheritance relationships in our representation by asserting that a *Eukaryotic*-*Cell* inherits a *Chromosome* and *Ribosome* from a *Cell*, and further, by specifying the inherited *Chromosome* as a *Eukaryotic*-*Chromosome*. We can capture such relationships if we rewrite A1 and A2 by using Skolemization, a well-known technique to approximate existential variables in the antecedent of an axiom [21]. With Skolemization, in an axiom of the form ∀ *Y*_1_ … *Y*_*n*_ ∃ *X* … *φ*, the existential variable *X* can be removed and replaced everywhere in *φ* with the function term *f*(*Y*_1_ … *Y*_*n*_), where *f* is a new function symbol that does not occur anywhere else in the axiom. The rationale for such a substitution is that, for any query, the original axiom is unsatisfiable if and only if the transformed axiom is unsatisfiable [21]. This implies that a query with an original axiom in the KB can be answered if and only if it can be answered when posed against the KB with the Skolemized version of the same axiom. However, from the point of view of logical entailment, the Skolemized KB is stronger than the original one, which is why we say that Skolemization only approximates existential quantification and is not equivalent to it. Skolemization of A1 and A2 enables referring to the Skolem functions introduced in them outside the scope of the existential quantifier. In the Skolemized versions of axioms A1 and A2 shown below, we can see that A4 refers to the Skolem functions introduced in A3.

Axiom A3:


Axiom A4:


The equality statement used in A4 proves to be a powerful tool that explicitly shows the inheritance relationship. In some cases, equality statements can be inferred. For example, if a cardinality constraint asserts that a *Cell* has exactly one *Chromosome*, then one can deductively conclude that the *Eukaryotic*-*Chromosome* must be the same as the inherited *Chromosome*. However, associating such constraints is incorrect in many situations, as is the case for a *Eukaryotic*-*Cell*.

More details about our approach to knowledge representation [22] and reasoning are available in previously published papers [23–25]. We have translated KB Bio 101 into multiple different formats including Web Ontology Language Version 2 (OWL2) functional^c^, answer set programming, and the Thousands of Problems about Theorem Proving syntax^d^. The translation into OWL2 is lossy, as it cannot fully capture the graph structures represented in the KB; the other translations are non-lossy. These translations are available through our website^e^, and an OWL version is available through BioPortal^f^.

### Upper ontology in AURA

AURA uses an upper ontology called *Component Library* or CLIB [26]. CLIB is a linguistically motivated ontology designed to support representation of knowledge for automated reasoning. CLIB uses four simple, upper-level distinctions: (1) *Entity* (things that are); (2) *Event* (things that happen); (3) *Relation* (associations between things); and (4) *Role* (ways in which entities participate in events).

A unique feature of CLIB is that it provides a vocabulary of actions for modeling biological processes. An *Action* is a subclass of *Event.* In CLIB, the class *Action* has 42 direct subclasses, with 147 subclasses in all. Examples of direct subclasses include *Attach*, *Impair*, and *Move*. Other subclasses include *Move*-*Through* (which is a subclass of *Move*) and *Break* (which is a subclass of *Damage*, which is a subclass of *Impair*). To ensure generality, these subclasses were developed by consulting lexical resources, such as WordNet [27]; the *Longman Dictionary of Contemporary English* [28]; and *Roget*’*s Thesaurus* [29].

CLIB provides semantic relationships to define the participants of an action. These relations are based on a comprehensive study of case roles in linguistics [30] and include **agent**, **object**, **instrument**, **raw**-**material**, **result**, **source**, **destination**, and **site**. (The syntactic and semantic definitions that we developed for these relations are available elsewhere [31].) As an example, we consider the definition of **raw**-**material**. The semantic definition of **raw**-**material** is any entity that is consumed as an input to a process. The syntactic definition of **raw**-**material** is either it is the grammatical object of verbs such as “to use” or “to consume”, or the word “using” precedes it.

CLIB also provides the vocabulary needed to define the relationships that exist between entities, and between entities and events, and to associate properties with both entities and events. For example, the most frequent relationships help define the structural relationships that exist between entities [32]. We use such relationships for representing structure: **has**-**part**, **has**-**region**, **material**, **element**, and **possesses**. We have developed detailed definitions and guidelines for their usage. For example, we say that *X***has**-**region***Y* if *Y* is a region of space or a *Spatial*-*Entity* defined only in relation to *X*. The complete definitions of the CLIB concepts and relationships are available online^g^.

**Figure 1:**
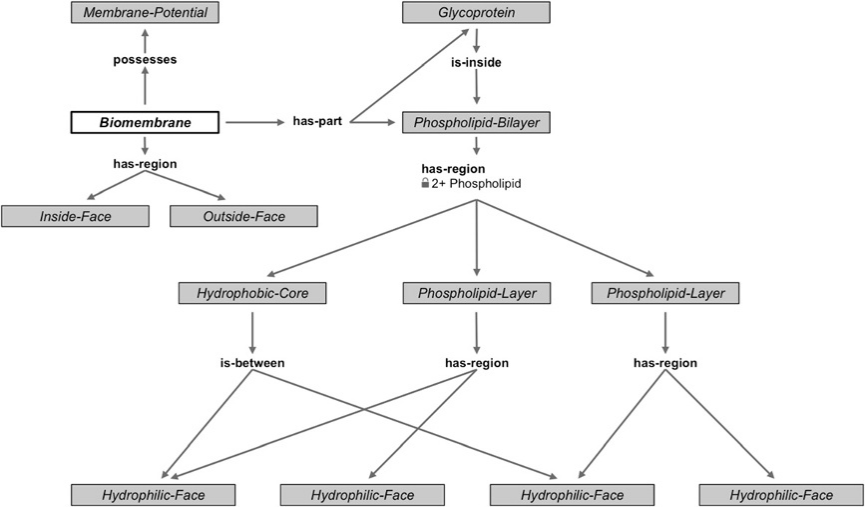
**A simplified view of the structure of** ***Biomembrane*** **represented in AURA.** The *Biomembrane* node (shown in white) is universally quantified, and every other node (shown in gray) is existentially quantified. We can read a portion of this figure as follows: for every instance of *Biomembrane*, there exists an instance of *Phospholipid*-*Bilayer* and an instance of *Glycoprotein* that are in **has**-**part** relationship to it, and further the instance of *Glycoprotein* **is**-**inside** the instance of *Phospholipid*-*Bilayer*. The usage of **has**-**part** and other relationships corresponds to instance-instance relationships [33].

As an illustration of the use of CLIB, in Figure 1, we show a simplified representation of the structure of a *Biomembrane*. From the representational point of view, the graph in Figure 1 represents an existential rule of the sort seen in axioms 1 and 2. In this figure, the node shown in white is universally quantified, and every other node, shown in gray, is existentially quantified. Therefore, we can read a portion of Figure 1 as follows: for every instance of *Biomembrane*, there exists an instance of *Phospholipid*-*Bilayer* and an instance of *Glycoprotein* that are in **has**-**part** relationship to it, and further the instance of *Glycoprotein***is**-**inside** the instance of *Phospholipid*-*Bilayer*. In the context of the relationships used in biomedical ontologies, our usage of **has**-**part** and other relationships corresponds to instance-instance relationships [33]. The arrows go from the first argument of a predicate to the second argument. For example, an arrow from *Biomembrane* to a *Phospholipid*-*Bilayer* labeled as **has**-**part** corresponds to the predicate **has**-**part**(*b*,*p*), where *b* is an instance of a *Biomembrane*, and *p* is an instance of a *Glycoprotein*.

**Figure 2:**
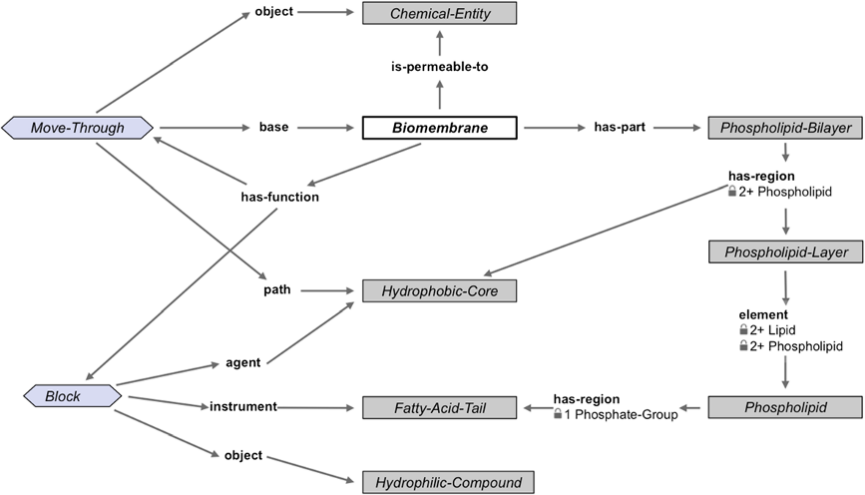
**Functions of** ***Biomembrane*** **.** The top half of this figure can be read as follows: every *Biomembrane* has a function to allow *Move*-*Through* of chemical entities that it is permeable to, and that this movement is through its *Hydrophobic*-*Core*, which is a region of its *Phospholipid*-*Bilayer*.

The numbers on some of the edges indicate cardinality constraints. For example, the instance of *Phospholipid*-*Bilayer* in Figure 1 has exactly two phospholipid layers that are in a **has**-**region** relationship to it. In Figure 2, we show the functions of a *Biomembrane*. A portion of this figure can be read analogously to Figure 1 as follows: for every instance of a *Biomembrane*, there exists a function *Block* in which the **agent** is a *Hydrophobic*-*Core*, the **object** is a *Hydrophilic*-*Compound*, and an **instrument** is a *Fatty*-*Acid*-*Tail*. More details about our representation of functions are available elsewhere [32].

### Reasoning in AURA

The KM system [14] provided the core reasoning services for AURA. KM’s reasoning combines description-logic-style classification [34] with backward chaining on rules. We extended KM’s basic reasoning with several higher-level reasoning methods to answer questions [24, 25]. AURA also contained a natural language processing interface that processed an input English question and converted it to a formal representation for evaluation by the reasoner [11]. We list below several abstract question templates, each followed by an example of its instantiation. A detailed formalization of different reasoning processes in AURA has been published elsewhere [24, 25]. To make this paper self-contained, we follow each question either by giving a high-level description of how that question was formalized or by specifying a logical query that could be evaluated by a general-purpose reasoner.

Q1. *What are the R of X*? (e.g., *What are the parts of a cell*?)

Q1 is a very common and basic form of query with numerous variations. Because the relevant knowledge to answer Q1 is in the form of axioms such as A1, the formalization of Q1 contains a premise that extends the KB to KB’ by creating a sample instance of *Cell*. For example, for the class *Cell*, and corresponding to the axiom A1, KB’ will contain the individual *c1*. By the application of A1, KB’ is further extended by adding *r1* and *ch1* such that they are instances of *Ribosome* and a *Chromosome*, respectively, and by adding the assertions (**has**-**part***c1 r1*) and (**has**-**part***c1 ch1*), which are conclusions derived by using A1. To answer Q1, we query for all literals matching (**has**-**part***c1* ?*x*), returning *c1* and *ch1* as answers. In more complex examples, query evaluation can involve inheriting information from super-classes and applying multiple rules.

Some instantiations of Q1 leverage the relation hierarchy in the KB. For example, “What is the structure of a cell?” Here, the word *structure* maps to the **has**-**structure** relationship in our ontology, which has four sub-relations: **has**-**part**, **has**-**region**, **material**, and **possesses**. For the values returned for each of these relationships, the system further retrieves spatial relationships to complete the structural description.

In more complex forms of Q1, further constraints on the values returned can exist. For example, consider: What does X do during Y? Assuming that we are interested in those steps such that X is a **raw**-**material**, those steps must also satisfy an additional constraint that they must be sub-steps of Y. Here, steps correspond to the phases of a process.

Q2. *What are the subclasses of X*? (e.g., *What are the subclasses of a eukaryotic cell*?)

Q2 is an example of a taxonomic query that queries for all subclass relationships for a class. In AURA, this query is answered by traversing the class-subclass hierarchy. Other queries similar to Q2 are: What are the super-classes of X? Is *X* a subclass of *Y*?

Q3. *How many X does a Y have for a relation R*? (e.g., *How many chromosomes does a human cell have as its part*?)

Q3 queries for the cardinality constraints on the **has**-**part** relationship for a human cell. AURA answers this query by a straightforward lookup of cardinality constraints.

Q4. *Describe X*? (e.g., *Describe a Cell*?)

To answer Q4, AURA computes all the facts known about a class. The facts about a class include taxonomic relationships (i.e., its super-classes and subclasses as computed in Q2); its relation values (as computed in Q1); and its cardinality constraints (as computed in Q3). AURA evaluates Q4 by issuing Q1, Q2, and Q3 as sub-queries, and then organizes the results in a concept description page.

Q5. *What is the difference*/*similarity between X and Y*? (e.g., *What is the difference*/*similarity between an integral protein and a peripheral protein*?)

AURA computes the answer to Q5 in three steps: (1) computing descriptions of *X* and *Y* as explained in Q4, (2) computing the similarities and differences between the two descriptions, and (3) then summarizing the results. We have described the details of the computations in a previous paper [25].

AURA supports more specific forms of Q5. For example: “*What are the structural differences between X and Y*?”; “*What is the difference between the size of X and size of Y*?”; etc.

Q6. *What is the relationship between X and Y*? (e.g., *What is the relationship between DNA and a gene*?)

Here, we are interested in computing how the individual instances of *X* and *Y* are related to each other. For example, how is an individual instance of a *DNA*-*Molecule* related to an individual instance of a *Gene*. One possible answer to this question is that a *DNA*-*Molecule* has as its part a *DNA*-*Strand*, which in turn, has as its part a *Gene*. To answer Q6, AURA first creates an individual instance of *X* and recursively computes its relation values (as in Q1) until it encounters an instance of *Y*. In general, multiple such relationships exist in the KB that should be ranked in the order of interest. AURA uses a variety of heuristics to limit the search process (for example, first searching the taxonomic relationships, preferring structural relationships, etc.).

AURA supports several questions that leverage the computation supported in Q6. Examples include: “*What are structural relationships between X and Y*?”; “*X is to Y as A is to what*?”; *and* “*Why is it important that X has property Y*?” To answer the question “*X is to Y as A is to what*?”, AURA first computes a path between *X* and *Y*, and then starting from *A*, traverses the same path to determine the answer [32]. An example formulation of the question “*Why is it important that X has property Y*?” is “*How does the selective permeability of membranes facilitates its function*?” To answer this question, AURA computes a path that begins from the permeability of a membrane and ends at the function of the *Membrane*, and that involves the relation **facilitates** [32].

We have implemented these reasoning methods in AURA and have extensively tested them. In the first stage of testing, we conducted a trial with students studying from *Inquire*. This initial test was done for the chapter on membranes. The results showed that the question templates were useful to the students, as the students using the facility achieved higher scores than the students studying from traditional methods, validating the choice of question templates [6]. Once the question templates were validated, we instantiated them for the first eleven chapters. The test suite for each chapter was spread across the content of the chapter and consisted of approximately 150 questions each. We executed the questions against AURA, and the domain experts rated the answers for correctness. From the 1,836 questions that we tested, the system correctly answered 1,540 questions, giving an overall correctness score of approximately 85%. These results showed a very high degree of system competence for answering questions. (For example, IBM’s Watson system that won the television game show *Jeopardy*! had a passing rate in mid-seventies [35].)

### Textbooks used for comparison

We chose to compare four textbooks spanning a range of breadth and depth of coverage (i.e., scope) based on the following rationale: choose one textbook comparable to *Campbell*, one textbook at a grade level lower, one textbook at a grade level higher, and one textbook at the advanced graduate level. Specifically, we used (1) *Raven*, which represents a textbook with a similar scope to *Campbell* [36]; (2) *Levine*, which offers both less breadth and depth than *Campbell*, and is used in a lower-division undergraduate, non-major course [37]; (3) *Alberts*, which has a narrower breadth, but a greater depth than *Campbell*, and is used in an upper-division undergraduate class in cell biology, and is considered a reference text for cellular and molecular biologists [38]; and (4) *Kandel*, which targets the specific field of neuroscience, and is therefore, narrower in breadth but has greater depth than *Campbell*, and also contains additional topics such as cognitive science^h^ [39]. *Kandel* is a textbook written for advanced undergraduates, graduate students, and medical students studying neuroscience, a specialized field that is largely biological, but also concerns itself with psychology and cognitive science. *Kandel* differs from the other textbooks in that different authors who are experts in their respective fields contributed most of the individual chapters. This approach may lead to a less-uniform treatment across the book than the other textbooks, which are each written by a small team of authors. Data comparing the relative lengths of these four textbooks is summarized in Table 1 below.

**Table 1:** **Data on page length and chapters in selected textbooks**

Textbook	Pages	Chapters	Pages/chapter
*Campbell*	1263	56	23
*Raven*	1298	57	23
*Levine*	1034	45	30
*Alberts*	1728	45	69
*Kandel*	1316	67	20

### Goals of research

We divided the high-level goal of investigating to what extent do our current process and methodology for capturing the semantics of textbook knowledge generalize to a range of life sciences textbooks into the following three more-specific questions: (1) To what extent is knowledge shared between the different textbooks? (2) To what extent can the same ontology be used to represent the knowledge found in different textbooks? (Based on our work with *Campbell Biology*, we were aware of many of CLIB’s limitations, especially because, from an AI perspective, fully capturing natural language text is an extremely difficult problem. Our goal here was to quantify the extent to which we could represent knowledge by using the existing CLIB vs. extending it to address any new requirements as we model different textbooks.) (3) To what extent can the questions of interest for a new textbook be answered by using the reasoning mechanisms already available in AURA? Because the foundational set of questions is expected to be similar in all domains, we expected good generality, but we wished to quantify it against each textbook.

## Methods

We now consider our methods for answering each of the three specific questions introduced in the previous section.

### Domain analysis

The goal of domain analysis is to answer the question: To what extent is knowledge shared between the different textbooks? More specifically, we were interested in understanding whether KB creation for each new book should start from scratch or some knowledge from one book could be shared from another. Answering this question for the topics that appear in one textbook but not in another is straightforward. Therefore, we selected the topics of action potential and membrane structure, which appeared in each of the four textbooks. The team undertook a coarse analysis of the selected material and selected a few paragraphs for detailed analysis. The team compiled information such as the length of coverage, the actual biological content covered, figures, and the type of language used for describing the material. Such comparison gave us insight into the commonality of knowledge across different textbooks, and that information guided us as to what extent we could share the domain-specific content across the KBs for different textbooks.

### Knowledge representation analysis

The goal of the knowledge representation analysis was to answer the question: To what extent can the same upper ontology be used to represent the knowledge found in different textbooks? Next, we give an overview of the AURA knowledge-engineering process that was the basis of the representation analysis, we provide an approach for dealing with subject matter consensus, and we introduce categories of representation requirements.

### AURA knowledge-engineering process

We used an already established knowledge-engineering process to represent the content of a textbook [31] as the basis of this analysis. This process has two distinct phases: (1) representation design and (2) knowledge encoding. For the representation-requirements analysis, we performed only the representation design phase, which includes the following three steps: (1) determining relevance: analyze each sentence in the textbook for its relevance for answering questions; (2) writing *universal truths* (UTs): for each relevant sentence, paraphrase it as a universally true statement about a specific entity or an event; and (3) developing action items for encoding: for each universally true statement, identify the concepts and relations that will be used for representing it.

We illustrate the above process by considering an example sentence: “*Many cells*, *including most prokaryotes*, *also produce a strong supporting layer around the membrane known as a cell wall*”. Multiple UTs can be derived from this sentence. One UT is: “*Many cells produce a cell wall*”. The use of word *many* is also indicative of the fact that there are some exceptions to this UT. To handle such exceptions, our knowledge-engineering process dictates that this statement should be further specialized for cells (for example, plant cells always produce a cell wall). Thus, the UT will be reformulated as “*All plant cells produce a cell wall*”.

Our general strategy to deal with exceptions is finding a class for which that statement is applicable as a universal truth. We ignore any exceptions that cannot be dealt with by using such a strategy. With the CLIB ontology, the UT under consideration will be represented by asserting that every *Plant*-*Cell* is an **agent** of a process called *Synthesis*-*of*-*Cell*-*Wall*, which has a **result** of *Cell*-*Wall* which **is**-**part**-**of** the *Plant*-*Cell*. Here, **agent**, **result**, and **is**-**part**-**of** are relations from the CLIB ontology. As a second example, consider the UT: “*Every plant cell has a cell wall that is a strong supporting layer*”. This UT will be represented by asserting that every *Plant*-*Cell***has**-**part** a *Cell*-*Wall* that **has**-**function** a *Support* that has an **object** the *Plant*-*Cell* itself, and has an **intensity** value of *strong*. Here, **has**-**function**, **object**, and **intensity** are relations in the CLIB ontology. As a final example, consider the following sentence: “*A protoplast is a plant cell without a cell wall*”. The UT for this sentence will be: “*Every protoplast is a cell without a cell wall*”. Clearly, the sentence fragment “every protoplast is a plant cell” cannot be universally true in our representation, because in that case, *Protoplast* will inherit all the properties of a *Plant*-*Cell* including a *Cell*-*Wall*. We will define *Protoplast* as a subclass of *Cell* in our class hierarchy. The relationship between a *Plant*-*Cell* and a *Protoplast* will be captured by other means.

Another central feature of AURA’s knowledge-engineering process is the division of labor between knowledge engineers and domain experts: the knowledge engineers have access to the full power of the representation language—which, as was explained earlier, is comparable to first-order logic with equality—but the domain experts create only new classes, declare classes to be disjoint, specify cardinality constraints, and, most importantly, author existential rules of the sort visualized in Figures 1 and 2.

### Achieving consensus among domain experts

Our approach to achieving consensus among the different domain experts working on the project is driven by the following observations: (1) Even for biological knowledge at the level of an introductory college course, no two textbooks are exactly the same. (2) A textbook such as *Campbell* has a large number of reviewers who are able to approve the content of the textbook. (3) Despite the differences in the textbooks, the students can be evaluated using a common test, and their answers can be rated. The key lessons that we drew from the textbook-authoring process is to aim for a process in which the project experts could review a representation and have an objective test for evaluating the knowledge in the system. We developed an extensive set of knowledge-engineering guidelines that prescribe how the domain experts should go about capturing textbook knowledge [31, 40]. Just as a textbook undergoes a review process, the representations undergo a review process that ensures an adequate application of the guidelines. This review does not mean that a representation meets an expert’s personal view on how the knowledge should be modeled, but rather ensures that the established encoding guidelines are adequately applied. Question and answer pairs stated in English provide a natural objective test to check the adequacy of the representation in the same way as students can be objectively tested on an exam.

### Inventory of representation requirements

The representation requirements can be put into two categories: (1) requirements that are already supported in CLIB and (2) requirement that are not currently supported. When we cannot model a universal truth in a straightforward manner by using the constructs available in the CLIB, we note this as a new KR requirement. The new KR requirements are strongly dependent on the state of CLIB at the time of the analysis. For answering the question of whether the same upper ontology could be used across multiple textbooks, however, the primary issue is the applicability of the representations supported in CLIB and the commonality of each new requirement across different textbooks.

KR requirements can arise due to the following reasons: (1) The knowledge can be represented by using the current features of the representation language and CLIB, but no established knowledge-engineering guidelines exist to handle it. We refer to the challenges arising due to this reason as *process issues*. (2) Representing the knowledge requires intervention from a knowledge engineer to extend the upper ontology. We refer to the issues arising due to this reason as *requiring knowledge*-*engineer support*. (3) Representing the knowledge is a topic of current and future research, and the current research has not yet been incorporated into the project. We refer to such issues as *requiring research and application*. We now give an inventory of the KR requirements that were encountered during the process, and we indicate into which of the above three categories each requirement fell.

#### Negative information

We say that a UT has a negative information KR issue if it cannot be modeled by using any of the four existing methods for handling negative information: (1) disjointness between classes, (2) cardinality constraints, (3) relations with negative meaning, and (4) negative values. As an illustration, consider the following sentence from *Raven*: “*Because these chains are nonpolar*, *they do not form hydrogen bonds with water*, *and triglycerides are not water*-*soluble*”. Here, we can state that a polar molecule is disjoint from a nonpolar molecule (to capture the nonpolarity), and we can assign a value of “insoluble” to the property **solubility**-**in**-**water**. In principle, one could introduce a slot with negative meaning (for example, **does**-**not**-**form**, or use a qualified number constraint on all *Create* processes in which *Nonpolar*-*Chains* participate that asserts that the **result** contains exactly zero *Hydrogen*-*Bond*s). However, no established methodology exists regarding which approach to use. Therefore, dealing with the example of negative information considered here is a process issue.

#### Missing relationships

We say that a UT cannot be expressed because of a missing relationship if the necessary relationship is missing from the vocabulary. An issue already known based on our work with *Campbell* is the lack of certain spatial relationships. As an illustration of this issue, consider the following sentence from *Raven*: “*Although the distribution of membrane lipids is symmetrical in the ER where they are synthesized*, *this distribution is asymmetrical in the plasma membrane*, *Golgi apparatus*, *and endosomes*”. Here, we need a new relation to capture asymmetrical distribution. Missing relationships require knowledge-engineer support.

#### Inability to state graded quantifiers

Recall that whenever the textbook uses words such as “many”, “most”, “typically”, etc., our KE strategy is to find a more-specific subclass for which the statement is universally true. This strategy breaks down when the textbook does not contain information about such a specific subclass. For example, consider the following sentence from *Levine*: “*Most prokaryotes and many eukaryotes have cell walls*”. The main difference between this sentence and the sentence: “*Many cells*, *including most prokaryotes*, *also produce a strong supporting layer around the membrane known as a cell wall*”, which we considered earlier, is the that *Levine* does not offer any specific examples of cells that do contain cell walls, so we cannot apply our KE strategy that worked for the earlier sentence. Whenever we encounter such a situation, we label it as an inability to state graded quantifiers, and it is a research and application issue.

#### Modeling biological models and reified statements

The textbooks frequently describe models and theories about natural phenomena. The statements about models are not universally true statements, but instead are contextual statements that hold true only in the context of that model. As an illustration, consider the following statement from *Alberts*: “*These regions cannot be identified in hydropathy plots and are only revealed by x*-*ray crystallography*, *electron diffraction* (*a technique similar to x*-*ray diffraction but performed on two*-*dimensional arrays of proteins*), *or NMR studies of the protein*'*s three*-*dimensional structure*”. Here, the presence of the regions is contextual to a particular set of techniques. Such knowledge can be captured in AURA, but the relevant guidelines have not been developed yet, and therefore, it is a process issue.

#### Property value comparison

A need frequently exists to compare property values. The CLIB ontology contains several comparison operators for properties, but we saw some examples where none of the existing operators were directly applicable to some sentences in the new textbooks. For example, consider the following sentence from *Raven*: “*However*, *at the end of each action potential*, *the cytoplasm contains a little more sodium and a little less K than it did at rest*”. Here, we need qualitative operators to capture relationships such as “little more” and “little less”. This issue requires knowledge-engineer support.

#### Causation

The notion of causality associated in the context of processes where causal relationships of events are of primary interest is already supported in CLIB. The textbook very often explains things by using the words such as “because”, “causes”, etc. We use the category label of causation to capture such issues as the current CLIB does not provide support to model such information. For example, consider the following sentence from *Alberts*: “*The shape and amphiphilic nature of the phospholipid molecules cause them to form bilayers spontaneously in aqueous environments*”. This KR requirement requires both research and application.

#### Disjunction

A need arises to capture two or more alternatives in a UT that cannot be modeled by another means. For example, consider the following sentence from *Alberts*: “*Hydrophilic molecules dissolve readily in water because they contain charged groups or uncharged polar groups that can form either favorable electrostatic interactions or hydrogen bonds with water molecules*”. This KR requirement requires both research and application.

#### Conditionality

Capturing a conditional statement in a UT that cannot be modeled by another means is sometimes necessary. Our general approach for capturing conditional statements has been using the class hierarchy. We create a new class, and the “if” part of the condition becomes a sufficient property for that class, while the “else” part of the condition becomes the necessary properties of that class. Such an approach works for most situations; but in some cases, it leads to unnatural classes, and thus is undesirable. For example, consider the following sentence from *Alberts*: “*This change of state is called a phase transition*, *and the temperature at which it occurs is lower* (*that is*, *the membrane becomes more difficult to freeze*) *if the hydrocarbon chains are short or have double bonds*”. Here the conditionality is between the temperature and the properties of hydrocarbon chains. If we model this knowledge by using sufficient properties, then creating unnatural classes, such as phase transition for short hydrocarbon chains, would be necessary. Handling this requirement is a process issue.

#### Possibility

Many sentences make statements of the form “A can B”, without necessarily stating that “A always does B”. We refer to the representation needs of such sentences as *possibility*. For example, consider the following sentence from *Alberts*: “*The free hydroxyl group contributes to the polar properties of the adjacent head group*, *as it can form hydrogen bonds with the head group of a neighboring lipid*, *with a water molecule*, *or with a membrane protein*”. Dealing with this KR requirement is a research and application. Initial steps in this direction could be undertaken by using research result on representing dispositions [41].

#### Data interpretation

In the advanced textbooks, figures are shown that contain representative data. The text then describes the form of the data and what conclusions either were or could be derived from this data. Thus, the figures are not just meant to illustrate a model but also to teach students how the actual data led to a set of conclusions. As an illustration, consider the following sentence from *Alberts*: “*In a normal unclamped axon*, *an inrush of Na* + *through the opened Na channels produces the spike of the action potential*; *inactivation of Na channels and opening of K channels bring the membrane rapidly back down to the resting potential*”. Dealing with this requirement is a research and application issue.

#### Science as a process

Particularly in *Kandel* and also in *Alberts*, many of the biological concepts are presented in the context of the process of science, i.e., scientists go through a process of testing, interpreting data, and developing hypotheses that are then tested again. For example, consider the following sentence from *Kandel*: “*A simple interpretation of these results is that the depolarizing voltage step sequentially turns on active conductance channels for two separate ions*: *one type of channel for inward current and another for outward current*”. Dealing with this requirement is a research and application issue.

#### Qualitative number constraint

Our current representation approach enables quantitative number constraints. We saw several examples in the textbooks where the constraint values are qualitative, and no other encoding approach sufficed. For example, consider the following example from *Raven*: “*Mammalian membranes*, *for example*, *contain hundreds of chemically distinct species of lipids*”. Dealing with this requirement requires knowledge-engineer support.

#### Mathematical reasoning

CLIB provides two different representations to facilitate mathematical reasoning: (1) simple qualitative relationships such as direct proportionality and (2) reasoning with mathematical equations. However, *Kandel* presents more complicated equations beyond CLIB’s current representational and reasoning capabilities. *Kandel* also includes derivations of mathematical formulas that cannot be represented by using current capabilities. For example, consider the following sentences from *Kandel*: “*When tetraethylammonium is applied to the axon to block the K*+ *channels*, *the total membrane current l*_*m*_, *consists of l*_*c*_, *l*_*v*_*and l*_*Na*_. *This outward current reaches a plateau that is maintained for the duration of the pulse* (*Figure nine*-*3B*)”. Dealing with this requirement requires knowledge-engineer support.

#### Vagueness/ambiguity

Advanced textbooks cover frontiers of our knowledge, and hence, this vagueness or ambiguity is not due to pedagogical presentation. However, it can lead to a universally true statement that is relevant but too vague to properly encode. These sentences are found across all textbooks, and seem to be more common in *Alberts*. (For example: “*Membrane attachment through a single lipid anchor is not very strong*, *however*, *and a second lipid group is often added to anchor proteins more firmly to a membrane*”.) Dealing with this requirement requires research and application.

#### Other issues

We use the KR category of *other issues* for representation problems that do not clearly fit into any of the previous category. For example, consider the following sentence from *Raven*: “*From this simple molecular framework*, *a large variety of lipids can be constructed by varying the polar organic group attached to the phosphate and the fatty acid chains attached to the glycerol*”. Here the author is trying to convey the salient variance between different phospholipids. Certain aspects of this knowledge are easily captured as sufficient properties, but that approach may not always be enough, especially to answer a question of the form “*How can you get different instances of a phospholipid*?” For the purposes of answering similarity and difference questions, and relationship questions, a representation based on sufficient properties is adequate.

### Reasoning requirements analysis

The goal of the reasoning requirements analysis was answering the question: to what extent can the questions of interest for a new textbook be answered by using the reasoning mechanisms already available in AURA? We wanted to confirm that as we move across textbooks, we would not have to develop new sets of reasoning methods for answering questions. To perform the analysis, the domain-expert team developed sample questions about membrane structure and action potential for each of the four textbooks. The overall guidance was to focus on the kinds of questions that a student studying from the book might have. The biologists had access to the examples of educationally useful questions that we had previously developed for *Campbell*. Some variability in the style and difficulty of questions potentially exists, because we did not have a mutual validation of question sets authored by different biologists. The possibility also exists that we biased their question-authoring effort by showing them the questions from the prior effort on *Campbell*. However, because the questions from the previous effort received extensive feedback from multiple teachers and students, we believe that they were a good guideline for this exercise. The domain-expert team and the knowledge-engineering team jointly analyzed the questions.

The questions stated in English needed to be translated into the question templates supported by the system. Such translation is done by AURA’s question-understanding module [42]. In many cases, the English statement of a question is not very helpful for determining the computation that must be performed in answering that question. For example, consider the question: “*How does the position of the gates in gated proteins cause the blocking of the movement of ions across the membrane*?” We can re-formulate this question as: “*What is the causal relationship between the position of the gates in gated proteins and the blocking of the movement of ions across the membrane*?” Another formulation of the same question is: “*How are the position of the gates in gated proteins and the blocking of the movement of ions across the membrane causally related*?” In AURA, both of these formulations will be handled by using Q6, in which we search for the causal relationships between the two entities in the question, and we expect the answer to be contained in the retrieved path. To develop such reformulations, the knowledge engineers must extensively rely on their knowledge of AURA to determine whether a given question in the corpus could be translated into one of the existing templates. This approach introduces some imprecision into the analysis, but this is unavoidable without undertaking the actual implementation.

## Results and discussion

We now consider the results of our analysis of domain knowledge, and representation and reasoning requirements, for the four textbooks.

## Results and discussion on domain knowledge analysis

We first analyze the two topics that we chose for comparison: action potential and membrane structure, and then offer conclusions based on the analysis.

In Table 2, we summarize data about the length of description of the different topics across the five textbooks. To the extent that different textbooks emphasize different levels of detail, the corresponding KBs need to match that level of detail. To make this observation concrete, we consider below specific example comparisons of content across the three textbooks.

**Table 2:** **Data on the length of description of action potential and membrane potential**

	Action potential			Membrane structure		
Textbook	Pages	Images	Sentences	Pages	Images	Sentences
*Campbell*	7	7	91	6	12	160
*Raven*	10	9	58	6	5	91
*Levine*	2	2	37	2	1	17
*Alberts*	14	14	20	12	18	270
*Kandel*	20	16	280	4	1	75

*Campbell* covers membrane structure in greater depth than *Levine*, *Raven*, or *Kandel*, but is limited in its description of the molecular structure of phospholipids. *Raven* and *Alberts* devote more detail to the molecular structure of phospholipids. *Levine* introduces lipids but has no mention of their more specific forms, such as glycolipids, which are mentioned in the other textbooks. In *Kandel*, membrane structure is not a major topic (it is more a topic in general biology than in neuroscience).

*Campbell* describes equilibrium potential by providing a definition and presenting an equation for the mathematical model known as the Nernst equation, along with two examples using this equation. *Raven* provides a similar amount of information to *Campbell*, but omits any examples using the Nernst equation. *Alberts* provides a definition, derives the Nernst equation, and shows several examples. *Kandel* provides the greatest breadth and depth for membrane potential, and devotes an entire chapter (Chapter 8) to the passive electrical properties of the neuron that are important for understanding the influence of neuronal structure and other properties on short and long-range signaling. *Kandel* also covers the contribution of different types of membrane channels to the signaling properties of different parts of the neuron.

Next, we consider the biological themes that occur inconsistently across our sample of textbooks: evolution, disorders/disease, scientific uncertainty, and animal models. *Campbell*, *Raven*, and *Levine* do not mention evolution in the context of action potential, but *Alberts* and *Kandel* discuss evolution of action potential function and structure of membrane proteins, respectively. Although *Campbell* and *Raven* omit discussion of disease, *Levine*, *Alberts*, and *Kandel* provide examples of diseases that affect normal functioning of action potentials. The presentation of scientific uncertainty also varies considerably across textbooks. *Raven* omits any mention of scientific uncertainty in the context of action potential, while *Campbell* and *Levine* simply report its existence. *Alberts* suggests that scientists will resolve uncertainty without exception, but *Kandel* presents scientific inquiry with respect to action potential as an iterative process with some degree of uncertainty. Animal models for the study of action potential are not described in *Levine*, and a single experimental model is described in both *Raven* and *Campbell*. Although *Kandel* describes a single experimental model, the giant squid axon, this text also emphasizes experimental techniques and their specific role in elucidating aspects of the action potential. *Alberts* describes multiple experimental models for the study of action potential.

The examples above suggest a great deal of commonality as well as differences in how different topics are described across the textbooks. For example, on the topic of membrane structure, the KB for *Levine* will contain far fewer terms than the other KBs (e.g., terms such as Glycolipid would need to be omitted.) Similarly, the KB for *Alberts* and *Raven* will provide a much more detailed account of phospholipid structure than the KB for *Campbell*. Similarly, while the Nernst equation will exist in all the KBs, the example associated with its use (as in *Alberts*), and a description of electrical properties (as in *Kandel*), will be specific to the KBs for those textbooks only. Differences in how to handle evolution, uncertainty, diseases, and animal models can have major repercussions in KB design.

Our analysis above suggests that a great deal of commonality across textbooks can be leveraged in creating a KB for each of them. At the minimum, the experience and representation approaches developed for one textbook can contribute toward a faster design of representations for a different textbook. Our analysis does not provide sufficient information about whether the domain-specific axiom writing for the textbook for a new KB should begin from scratch or should reuse the axioms from the previous ones. Clearly, some reuse should be possible, but the extent of reuse and its cost effectiveness is an open question. Further, our analysis provides concrete examples of where the textbooks have substantial differences requiring representation design that is specific to that textbook.

### Results and discussion on knowledge representation requirements

In Table 3 below, we summarize all the KR issues along with the textbooks for which the issue was encountered. The column labeled as “New issue” indicates an issue that we have not encountered or so far addressed in our work with *Campbell Biology*.

**Table 3:** **Observed knowledge representation issues**

Category of KR issue	Occurs in textbooks				Occurs in Campbell?	New issue?
	***Levine***	***Raven***	***Alberts***	***Kandel***		
Negative information		x	x	x	x	No
Spatial relation	x	x	x	x	x	No
Missing slot (other than spatial relation)		x	x	x	x	No
Inability to state graded quantifiers	x	x	x		x	No
Biological models and reified statements	x	x	x	x	x	No
Property-value comparison		x			x	No
Causation			x		x	No
Disjunction	x		x		x	No
Conditionality			x		x	No
Possibility			x		x	No
Data interpretation			x	x		Yes
Science as a process			x	x	x	No
Qualitative number constraint		x			x	No
Mathematical reasoning				x	x	No
Vagueness/ambiguity		x	x	x	x	No
Other		x			x	No

In Table 4 below, we show the results that indicate the number of UTs for each of the textbooks that could not be adequately represented for the topic of action potential. For each UT that could not be represented, we identify a knowledge representation category to indicate the nature of requirement. We next explain these results for each of the textbooks.

**Table 4:** **KR requirements by category**, **for the topic action potential**

Category of KR/KE issue	Number of UTs affected (%)			
	***Levine***	***Raven***	***Alberts***	***Kandel***
Negative information	3 (8%)	6 (6%)	3 (2%)	
Spatial relation			3 (2%)	
Missing slot (other than spatial relation)	4 (8%)	3 (3%)	3 (2%)	6 (7%)
Inability to state graded quantifiers			1 (1%)	2 (2%)
Modeling biological models and reified statements			1 (1%)	3 (3%)
Property-value comparison		3 (3%)		
Causation				
Disjunction	1 (3%)			
Conditionality				
Possibility			11 (8%)	
Data interpretation			11 (8%)	4 (4%)
Science as process				8 (9%)
Qualitative number constraint				
Mathematical reasoning				3 (3%)
Vagueness/ambiguity		1 (1%)	2 (1%)	
Other				
**Total**	**8** **(21%)**	**13** **(13%)**	**35** **(26%)**	**26** **(30%)**

For *Levine*, approximately 20% of UTs for action potential had new KR requirements. In addition, negative information that could not be adequately encoded occurred for action potential, and we encountered one instance of disjunction that could not be adequately encoded. The issues of lack of specificity and models did not arise for action potential for this text. For the *Raven* textbook, 13% of the UTs were problematic. For *Alberts*, approximately 25% of UTs presented new KR requirements for action potential. In addition, the new KR requirement of data interpretation arose. In *Kandel*, approximately 30% of UTs presented new KR requirements. Thus, like *Levine* but unlike *Raven* and *Alberts*, *Kandel* presented proportionally more issues for action potential. For example, data interpretation issues and science as process issues arose frequently. Further, for action potential, *Kandel* contained sentences outside AURA’s current mathematical representation and reasoning capabilities.

In Table 5 below, we show our results of how well we could represent the topic of membrane structure for each of the four textbooks. Detailed explanations follow.

**Table 5:** **KR issues by category**, **for the topic membrane structure**

Category of KR/KE issue	Number of UTs affected (%)			
	***Levine***	***Raven***	***Alberts***	***Kandel***
Negative information		3 (1%)	17 (7%)	2 (2%)
Spatial relation	3 (4.5%)	17 (7%)	15 (6%)	2 (2%)
Missing slot (other than spatial relation)		6 (2.5%)	8 (3%)	4 (4%)
Inability to state graded quantifiers	6 (9%)	28 (12%)	10 (4%)	
Modeling biological models and reified statements	1 (1.5%)	19 (8%)	1	6 (7%)
Property-value comparison				
Causation			2 (1%)	
Disjunction			1	
Conditionality			5 (2%)	
Possibility			3 (1%)	
Data interpretation				
Science as process				2 (2%)
Qualitative number constraint		1		
Mathematical reasoning				
Vagueness/ambiguity		2	15 (6%)	
Other		3 (1%)		
**Total**	**10** **(15%)**	**79** **(33%)**	**77** **(31%)**	**16** **(18%)**

For *Levine*, we encoded approximately 85% of UTs without any facing any new requirements. The most common new KR requirements were missing relations (namely, spatial relations), and the inability to identify a sufficiently specific concept, as illustrated in the earlier example. *Raven* exhibits a greater percentage and breadth of new KR requirements than *Levine*. Nearly 35% of UTs did have new KR requirements. The most common KR requirements were, again, specificity of concepts and missing slots. A common requirement for *Raven* was representing biological models. *Raven* (and the other textbooks) had several examples of negative information of a form that cannot be represented with AURA’s current capabilities. *Alberts* has a similar percentage of new KR requirements to *Raven* and a greater breadth. Again, more than 30% of UTs posed some new KR requirement. Further requirements come from conditionality, causation, and possibility. Because *Alberts is a* research-oriented textbook, it describes topics at the limit of current biological knowledge. This leads to the greater number of UTs with the KR issues of vagueness compared to other textbooks. For *Kandel*, more than 80% of UTs were encoded without facing any new KR requirement, and no new requirements arose that did not arise for another textbook, except the need to represent knowledge about science as a process. Hence, in terms of number of issues, *Kandel* proved amenable to our KE process despite its more advanced nature.

Let us now consider how these results address the question: to what extent can the same upper ontology be used to model knowledge across a range of life science textbooks? The results in Table 3 suggest that all the requirements that were identified for the new textbooks, with the exception of data interpretation, were also requirements for *Campbell*. This finding is strong evidence in support of the claim that if these requirements were supported in an upper ontology, such ontology would be applicable across multiple textbooks. From Table 3, we also see that spatial relationships and biological models are the requirements that occur most uniformly across the textbooks, followed by negative information, graded quantifiers, and science as a process. These constitute high-priority areas for extending the CLIB ontology.

From Tables 4 and 5, we see that the existing upper ontology enabled us to capture at least 67% of all the UTs across all topics and across all the textbooks. In some cases, the coverage was as high as 87%. Based on these results, we can conclude that CLIB already provides a good foundation for representing knowledge across the range of life science textbooks considered here.

### Results and discussion on reasoning requirements

Recall that our high-level question regarding reasoning requirements was: To what extent can the questions of interest for a new textbook be answered by using the reasoning mechanisms already available in AURA? We gave an overview of the current questions supported in an earlier section.

To answer the above question, we assembled a suite of new questions for each of the four textbooks and put them into two different categories: (1) answerable with existing system capabilities, or minor extensions of them, supposing that the requisite concepts are encoded; and (2) require new reasoning capabilities, or major extensions of existing capabilities, or beyond anticipated feasible reasoning, or contingent on significant new research. We will now present the results of our analysis and will illustrate the questions that fall into each of these categories.

In Table 6 below, we summarize the overall analysis of questions about action potential and membrane structure.

**Table 6:** **Reasoning requirements analysis for action potential and membrane structure**

	Action potential			Membrane structure		
Textbook	Questions	Existing	Research	Questions	Existing	Research
*Raven*	16	12	4	21	17	4
*Levine*	53	43	10	32	25	7
*Alberts*	19	16	3	48	47	1
*Kandel*	50	43	7	29	26	3
Total	138	114	22	130	115	15

The column labeled as *Questions* indicates the total number of questions considered in the analysis. The column labeled as *Existing* indicates the number of questions that could be handled by using existing capabilities in AURA, and the column labeled as *Research* indicates the number of questions that cannot be handled by the current capabilities in AURA and that require further research.

Across the four textbooks on average, we observe that for action potential, approximately 85% of the questions are category 1 (existing capability), and 15% are category 2 (representational extension or significant reasoning requirements). For membrane structure, nearly 90% of the questions are category 1 (existing capability), and 10% are category 2 (representational extensions or significant reasoning requirements).

From each of the four textbooks, we now give example question forms that could be answered by using the existing capability. For each question form, we give an example question, its model answer if provided, and a reformulation of the question. Because each of these question forms can be answered by using the existing capability (or a minor extension of it) through the given reformulations, new question templates are not required.

*● Question template in English*: *What is the role of X* (*in context Y*)?

*○ Example instantiation from the sample question set*: “*In the equivalent electrical circuit model*, *what cellular element serves as the resistor*?” [*Kandel*]

*● Question template in English*: *Why is it important that X has property Y*?

*○ Example instantiation from the sample question set*: “*Why is it important that membranes are selectively permeable*?” [*Levine*]

*○ Reformulate as*: “*How does the selective permeability of membranes facilitate its function*?”

*● Question template in English*: *What kinds of X are common in Y*?

*○ Example instantiation from the sample question set*: “*What kinds of lipids are common in cell membranes*?” [*Levine*]

*○ Reformulate as*: “*What are the lipid parts of a cell membrane*?”

*● Question template in English*: *What does X do during Y*?

*○ Example instantiation from the sample question set*: “*What is the sodium potassium pump doing during an action potential*?” [*Levine*]

*○ Reformulate as*: “What does sodium potassium do during an action potential?”

*● Question template in English*: *What features of X affects its role in Y*?

*○ Example instantiation from the sample question set*: “*What features of the voltage*-*gated sodium channel affect its role in an action potential*?” [*Raven*]

*○ Reformulate as*: “*What is the relationship between a voltage*-*grated sodium channel and action potential*?” (This reformulation is approximate as it does not specifically ask for the relationship to role in the action potential.)

We now consider example questions that require new question templates. For each, we give an example question template and its instantiation.

*● Question template in English*: *What is the importance of X*?

*○ Example instantiation from the sample question set*: “*What is the importance of plasma membrane fluidity*?” [*Alberts*]

*● Question template in English*: *What aspects of X can be seen by Y*? (where Y is a inspection technique, instrument, or process)

*○ Example instantiation from the sample question set*: “*What aspects of the plasma membrane can be seen by transmission electron microscopy* (*TEM*)?” [*Raven*]

*● Question template in English*: What properties of X contribute to the property Z of Y?

*○ Example instantiation from the sample question set*: “*What characteristic of phospholipids contributes most to the membrane*-*forming properties of these molecules*?” [*Alberts*]

*● Question template in English*: *Given that X does Y*, *why does Z also not do Y*?

*○ Example instantiation from the sample question set*: “*Given that the sodium*-*potassium pump results in a net transport of positive ions from the inside of the cell to the outside*, *why don*'*t negative ions also leave the cell to balance out the charge difference*?” [*Raven*]

*● Question template in English*: *Which strategy does X use to achieve Y*?

*○ Example instantiation from the sample question set*: “*Vertebrate systems generally rely on what adaptive strategy for increasing the rate of axonal conduction*?” [*Kandel*]

The quantitative results in Table 6 support the conclusion that a large fraction of the questions in the test suite assembled by the domain experts (greater than 85%) for a new textbook could be answered by using the reasoning mechanisms already available in AURA. This finding is an extremely positive result that attests to the generality of the already-implemented reasoning mechanisms. However, we would like to emphasize that given the bias introduced by exposing the domain experts to the existing capabilities, we should not take these results to conclude that the existing capabilities could answer greater than 85% of all possible questions posed against these textbooks. These results are applicable to only to a specific style of educationally useful questions that have been found helpful in our work on the intelligent textbook. These results show that such questions have a high degree of generality and applicability across the range of textbooks considered in this analysis.

### Comparison to related work and broader impacts

In this section, we relate the work presented here to related efforts in modeling knowledge by using OWL and other biomedical ontology development efforts. We also comment on how our work can be exploited by others.

Most of the representation features used in AURA are also found in OWL (for example, classes; class-subclass relationships; disjoint statements between classes; domain; range; qualified number constraints; etc.). Our work to capture graph-structured knowledge of the sort illustrated in axioms A1–A4 is closely related to recent efforts to extend OWL to capture graph-structured descriptions [17]. Others have recognized the need to support graph-structured descriptions to capture chemical structures [16], and active research is underway to address it [17–20]. KB Bio 101 already contains several hundred examples of complex concepts that utilize such graph-structured representation [13], such as the ones shown in Figures 1 and 2. One possible technique to achieve decidable reasoning in a KB with graph-structured descriptions is to avoid certain kinds of cyclical dependence among concepts [17], but no empirical evaluation exists of such a technique on a realistic, large-scale dataset. KB Bio 101 is an excellent candidate data set for undertaking such evaluation. More generally, KB Bio 101 can be used as a dataset for testing techniques for ontology modularization, ontology mapping, ontology evaluation, development of ontology design patterns, etc.

In several prior publications, we related the representations supported in CLIB with the ones adopted for biomedical ontologies (for example, in [32], we describe our representation for structure and function; in [43], we describe representation of roles; and in [44], we describe the representation of genetic entities). Gene Ontology or GO [45] is a closely related community-wide effort that supports molecular-level and cellular-level representations for gene function. Because life science textbooks cover knowledge at organismal, species, and population levels, the scope of knowledge represented in KB Bio 101 is much broader than the knowledge represented in GO.

A unique feature of our ontology that none of the other biomedical ontologies supports is a vocabulary of process classes (e.g., *Move*, *Attach*, *Release*, etc.) and their detailed definitions using semantic relationships (e.g., **agent**, **object**, **source**, **destination**, etc.). Due to lack of such vocabulary, ontologies such as GO define functions using only textual strings and functions are not compositionally defined to capture their complete meaning. The CLIB approach to modeling processes and their participants can be readily exploited by biomedical ontologies to achieve a much greater depth of knowledge capture for biological functions.

A driving use case for GO, and a major contributor to its success, has been its use in annotation projects. The question templates Q1–Q6 introduced in our work can provide another compelling use case for exploiting GO and other biomedical ontologies. Although Q1–Q6 were driven by the needs of education applications, similar reasoning can be useful for biological discovery applications such as [46].

Many educational innovations begin at the graduate level, and slowly find their way to undergraduate and precollege-level education. Therefore, perhaps, the most impactful way to exploit this work is using it as an example to start incorporating biomedical ontologies into undergraduate and high-school-level curricula for life science education. Future life sciences graduates will need to routinely use ontology resources, and some of these graduates will need to help create new ones. However, ontologies are not yet a standard part of the life sciences curriculum. Students are not normally exposed to ontologies unless they enter a graduate program in bioinformatics. We believe that now is the time to begin making training in formal languages and their ontological commitments an integral part of the life sciences curriculum. Wider use of ontologies in the life sciences will lead to better understanding and communication of knowledge by teachers and students. Such explicit usage of ontologies is different from the methods used by search tools such as Google, which are excellent for retrieval but do little to improve our understanding of the subject matter.

## Conclusions

We present our conclusions for each of the three major analyses presented here: (1) domain knowledge requirements, (2) knowledge representation requirements, and (3) reasoning requirements. We acknowledge at the outset that our conclusions are based on the data gathered for the topics of action potential and membrane structure. Our generalized conclusions are based on the hypothesis that these data could be generalized to other biological topics in the textbook.

The results of our **domain requirements** analysis show that, as expected, the *Levine* textbook, which is aimed at a lower instructional level than *Campbell*, presents material from a more general perspective, omitting details that *Campbell* and *Raven* include. Likewise, the textbooks aimed at a higher instructional level than *Campbell* present details that *Campbell* does not. The breadth and depth of coverage for action potential and membrane structure appear most similar between *Campbell* and *Raven*. We also found that the textbooks for instruction levels higher than *Campbell* and for a specific field of biological sciences do not cover the broad range of knowledge in *Campbell* but instead rely on *Campbell*’s prerequisite biology knowledge, and build on a fraction of this foundation. For example, *Kandel* provides considerably less breadth on the topic of membrane structure compared to *Campbell*. The details of membrane structure are likely omitted from *Kandel* because the authors deem such information as prerequisite or not germane to the sub-discipline of neuroscience. Our results suggest that the modeling effort invested in representing any of these books will reduce the cost of doing additional books. Because the considered textbooks vary in detail, and in their choice of the aspects of biology knowledge to emphasize, the KB for each of these textbooks also must be customized and made specific to that particular textbook.

The results of our **knowledge representation requirement** analysis showed that the knowledge-engineering process used for *Campbell* appears to be effective across the range of considered textbooks. We encountered no major surprises regarding modeling issues: most of the issues that we saw in these textbooks also exist in some form for *Campbell*. We confirmed that the studied textbooks that were written for the same grade level (i.e., *Campbell* and *Raven*) were comparable in their knowledge content and representation requirements. We found an increase in presentations of theories, models, and history in the higher-level textbooks, which is expected as the textbooks for the higher grade levels are closer to the frontiers of knowledge. For example, *Kandel* describes the experiments that are used to test a model or hypothesis, and the reasoning process that was used to support or refute that model. Our overall conclusion was that our existing representation tools are applicable for modeling knowledge across the range of considered textbooks, and that the new requirements identified here will have broad applicability to multiple textbooks.

Based on the **reasoning requirements** analysis, we can conclude that a majority of the biologist-authored, educationally useful questions for each of the textbooks can be adequately addressed by using extensions to AURA’s current capabilities. This assertion is true because all the textbooks had the same foundational set of questions and were all based on the same foundational biology. The reasoning patterns of relationship questions and comparison questions seem to be directly applicable across multiple textbooks. We also found that the answers for one textbook may contain vocabulary or detail that is unexpected at a different grade level. For example, *Levine* does not use the term “phospholipid bilayer”. In *Kandel* and *Alberts*, most answers are with respect to models and cannot be considered as universally true. We definitely cannot conclude that the existing question templates are adequate for the space of all questions that the readers of each of the textbooks might want to ask. Our previous work with *Campbell* also showed us that the existing templates are inadequate for capturing all the reasoning patterns.

A possible way forward is aligning the presented representations and approach with the methods that are already commonplace in biomedical research, and then start incorporating those representations in life sciences textbooks. As an example, consider the representation for *Kandel*. Especially for a field as broad as neuroscience, different groups will need to be engaged for different parts of a text like *Kandel*. In fact, in this textbook, different experts author different chapters to ensure that the content aligns with current thinking in the field. Efforts are underway through projects like the International Neuroinformatics Coordinating Facility^i^, the Blue Brain Project^j^, and the Neuroscience Information Framework^k^ to create a semantically unified body of broad neuroscience knowledge. When textbook knowledge is complemented with resources like these, the enhanced version is not only useful for biomedical research but can also serve as a valuable education tool.

Undertaking textbook knowledge representation as proposed here will profoundly shift the way we think of life science education. The semantic representations would serve as a conceptual mathematics that computers could rigorously reason over. Exposure to such representations as part of a life science education will likely instill graduates with an increased level of rigor in learning and working with biological concepts. The time is now ripe to introduce these techniques at all levels of biology education, so that students are well prepared for the computational thinking [47] that is both so vital to practitioners in today’s knowledge economy and indispensable for researchers pursuing advanced biomedical discoveries.

## Endnotes

^a^http://www.aaaivideos.org/2012/inquire_intelligent_textbook/

^b^Almost every universally true statement in biology has an exception. For example, there are eukaryotic cells that do not have a nucleus.

^c^http://www.w3.org/TR/owl2-syntax/

^d^http://www.tptp.org

^e^http://www.ai.sri.com/~halo/public/exported-kb/biokb.html

^f^https://bioportal.bioontology.org/ontologies/AURA

^g^http://www.ai.sri.com/~halo/public/clib/20130328/clib-tree.html

^h^The 5th edition of *Kandel* appeared when our study was underway.

^i^http://incf.org

^j^http://bluebrain.epfl.ch

^k^http://neuroinfo.org

## Authors’ information

Dr. Vinay K. Chaudhri is a program director in the Artificial Intelligence (AI) Center at SRI International. His research focuses on the science and engineering of large knowledge base systems and spans knowledge representation and reasoning, question answering, knowledge acquisition, and innovative applications. His most recent work has been on creating an intelligent textbook in biology that answers a student’s questions and leads to significant learning gains. He has co-edited a volume on the Theory and Application of conceptual modeling, and two special issues of AI Magazine — one on Question Answering Systems, and another on Application of AI to Contemporary and Emerging Education Challenges. He has taught a course on Knowledge Representation and Reasoning at Stanford University. He holds a Ph.D. in Computer Science from University of Toronto where he was a Connaught Scholar. He also holds a Masters in Industrial and Management Engineering from Indian Institute of Technology Kanpur, and a Bachelor’s degree in Mechanical Engineering from National Institute of Technology, Kurukshetra. He is a senior member of AAAI.

Daniel Elenius is a Computer Scientist at the Computer Science Lab at SRI International. He holds an MS in computer science and engineering from Linköping University, Sweden. He has developed several reasoning systems, including a policy reasoner for the DARPA neXt Generation (XG) program, a probabilistic fault propagation analysis tool for the DARPA META program, and a system that reasons about hierarchical tasks and resource assignments for the DoD ONISTT and ANSC projects. His research interests include automated reasoning, knowledge representation, and the semantic web.

Andrew Goldenkranz is a biology teacher at Monta Vista High School in Cupertino, California. He has helped position the *Inquire* application (an iPad app for AURA) so that it is useful for teaching students studying from *Campbell Biology*.

Allison Gong studies marine biology, particularly invertebrate zoology, and teaches biology at the community college and university levels in California. She teaches marine biology, zoology, and evolution to science majors and science-phobes alike. Her interests in marine biology focus on marine invertebrate life histories, larval biology, and ecology of the rocky intertidal. She holds a PhD in biology from the University of California.

Maryann E. Martone received her BA from Wellesley College in biological psychology and her PhD in neuroscience in 1990 from the University of California, San Diego, where she is currently a professor in the Department of Neuroscience. She is the principal investigator of the Neuroinformatics Framework project, a national project to establish a uniform resource description framework for neuroscience. Her recent work has focused on building ontologies for neuroscience for data integration. She has completed her tenure as the US scientific representative to the International Neuroinformatics Coordinating Facility (INCF), where she still heads the program on ontologies. MM recently joined FORCE11, an organization dedicated to advancing scholarly communication and e-scholarship, as Executive Director.

William Webb is an expert in wildlife biology and has taught biology courses to community college students for five years across multiple campuses. Dr. Webb has community college teaching experience in diverse topics within biology, including general education courses such as general biology and health science, in addition to major’s courses such as human anatomy and physiology and animal biology. He holds a PhD in wildlife science from the University of Washington.

Neil Yorke-Smith is an assistant professor at the American University of Beirut, Lebanon and a visiting scholar at St Edmund’s College, Cambridge. His research interests include intelligent agents, planning and scheduling, constraint-based modeling, intelligent user interfaces, and their real-world application to managerial decision-making. He holds a PhD in optimization from Imperial College London, UK.

## Abbreviations

AURA: Automated User Centered Reasoning and Acquisition System
CLIB: Component Library
GO: Gene Ontology
KB: Knowledge Base
KE: Knowledge Engineering
KM: Knowledge Machine
KR: Knowledge Representation
OWL: Web Ontology Language
UT: Universal Truth.
